# The synergistic role of ATP‐dependent drug efflux pump and focal adhesion signaling pathways in vinorelbine resistance in lung cancer

**DOI:** 10.1002/cam4.1282

**Published:** 2018-01-10

**Authors:** Takao Nakanishi, Toshi Menju, Shigeto Nishikawa, Koji Takahashi, Ryo Miyata, Kei Shikuma, Terumasa Sowa, Naoto Imamura, Masatsugu Hamaji, Hideki Motoyama, Kyoko Hijiya, Akihiro Aoyama, Toshihiko Sato, Toyofumi F. Chen‐Yoshikawa, Makoto Sonobe, Hiroshi Date

**Affiliations:** ^1^ Department of Thoracic Surgery Graduate School of Medicine Kyoto University Kyoto Japan; ^2^ Department of Thoracic Surgery Japanese Red Cross Wakayama Medical Center Wakayama Japan; ^3^ Institute for Advancement of Clinical and Translational Science Kyoto University Hospital Kyoto Japan; ^4^ Department of Thoracic Surgery Kobe‐City Nishi‐Kobe Medical Center Kobe Japan

**Keywords:** ATP‐Binding Cassette Subfamily B Member 1, chemotherapeutic resistance, focal adhesion pathway, non–small cell lung cancer, saracatinib, Src family kinase (SFK), vinorelbine

## Abstract

The vinorelbine (VRB) plus cisplatin regimen is widely used to treat non–small cell lung cancer (NSCLC), but its cure rate is poor. Drug resistance is the primary driver of chemotherapeutic failure, and the causes of resistance remain unclear. By focusing on the focal adhesion (FA) pathway, we have highlighted a signaling pathway that promotes VRB resistance in lung cancer cells. First, we established VRB‐resistant (VR) lung cancer cells (NCI‐H1299 and A549) and examined its transcriptional changes, protein expressions, and activations. We treated VR cells by Src Family Kinase (SFK) inhibitors or gene silencing and examined cell viabilities. ATP‐binding Cassette Sub‐family B Member 1 (*ABCB1*) was highly expressed in VR cells. A pathway analysis and western blot analysis revealed the high expression of integrins *β*1 and *β*3 and the activation of FA pathway components, including Src family kinase (SFK) and *AKT*, in VR cells. SFK involvement in VRB resistance was confirmed by the recovery of VRB sensitivity in *FYN* knockdown A549 VR cells. Saracatinib, a dual inhibitor of SFK and ABCB1, had a synergistic effect with VRB in VR cells. In conclusion, ABCB1 is the primary cause of VRB resistance. Additionally, the FA pathway, particularly integrin, and SFK, are promising targets for VRB‐resistant lung cancer. Further studies are needed to identify clinically applicable target drugs and biomarkers that will improve disease prognoses and predict therapeutic efficacies.

## Introduction

Lung cancer remains the leading cause of cancer death in western countries 1. Recent advances in advanced non–small cell lung cancer (NSCLC) therapy have focused on selective inhibitors that target driver mutations or genes that are critical to tumor growth and proliferation; this targeted therapy has led to dramatic clinical responses 2, 3. However, the conventional chemotherapeutic regimen continues to be used, particularly as a postoperative adjuvant chemotherapy, because adjuvant epidermal growth factor receptor (EGFR) tyrosine kinase inhibitor (TKI) has shown no survival benefit 4. As a postoperative adjuvant therapy, cisplatin plus vinorelbine (VRB) is the standard regimen because adjuvant cisplatin plus VRB shows a superior survival benefit in subgroup analyses 5. However, the postoperative 5‐year survival rates for pathologic stage II–IIIA patients are unsatisfactory, at 33–61% 6 without adjuvant therapy and 42–52% in a group that received adjuvant chemotherapy while lacking a residual tumor 7. Adjuvant chemotherapy has a modest effect toward prolonging survival, with an absolute 5‐year overall survival improvement ranging from 4 to 15% 8, whereas the response rate to cisplatin plus VRB or cisplatin plus paclitaxel is 25–28% in advanced NSCLC 9.

Drug resistance, whether intrinsic or acquired, is believed to underlie treatment failures in over 90% of patients with metastatic cancers 10. Multiple factors affect drug sensitivity. Although drug efflux transporters from the ATP‐binding cassette (ABC) family 11, 12 and class III *β*‐tubulin 13 are reportedly involved in VRB resistance, their validities as essential factors remain controversial 14. To establish more effective therapies, it is essential to elucidate key resistance pathways. Combination therapy with cytotoxic drugs and molecular target drugs that inhibit the resistance mechanism should be potent candidates for overcoming drug resistance and prolonging overall survival.

Focal adhesion (FA) pathways, particularly integrins and Src family kinase (SFK), play important roles in cancer cell survival, invasion, proliferation, and drug resistance 15, 16, 17. Although their roles in drug resistance in lung cancer are mainly reported in relation to EGFR TKIs 18, resistance mechanisms for cytotoxic drugs may also be affected by these focal adhesion signals.

To elucidate the mechanism of VRB resistance and identify effective drugs in VRB‐resistant cancer cells, we examined gene expression and protein phosphorylation in parental versus induced VRB‐resistant (VR) lung cancer cell lines. This report shows that ATP‐binding Cassette Sub‐family B Member 1 (ABCB1) and focal adhesion‐related proteins, particularly SFK and integrin *β*3, may be promising targets for overcoming VRB resistance.

## Materials and Methods

### Cell culture

Two human non–small cell lung cancer cell lines, NCI‐H1299 and A549, were maintained in the American Type Culture Collection (ATCC)‐recommended medium (RPMI 1640 or Dulbecco's modified Eagle's medium (Sigma‐Aldrich, St. Louis, MO)) supplemented with 10% fetal bovine serum (HyClone, Thermo Fisher Scientific K. K. Kanagawa, Japan) in standard culturing conditions (5% CO_2_, 100% humidity, 37°C). Mycoplasma negativity was confirmed for each cell line before use. VRB‐resistant cell lines were established using graded VRB concentration increases up to 20–100 times the initial concentration as previously described 12. During this process, the cell lines were moved into CELLBANKER 1 (Zenoaq, Koriyama, Japan) at each resistant stage (H1299 weak resistant, cultured in 5 nmol/L VRB; H1299 moderate resistant, 50 nmol/L VRB; H1299 VR strong resistant, 500 nmol/L VRB; A549 VR, 100 nmol/L VRB) and stored in liquid nitrogen until further use.

### Compounds

VRB, cisplatin, paclitaxel, docetaxel, and etoposide were purchased from Wako Pure Chemical Industries (Osaka, Japan). The SFK inhibitor, dasatinib, was purchased from Focus Biomolecules (Plymouth Meeting, PA), and saracatinib was purchased from Selleck Chemicals (Houston, TX). The ABCB1 inhibitor, tariquidar, was purchased from Toronto Research Chemicals Inc. (Toronto, ON, Canada). Cilengitide (integrin *α*v*β*3 inhibitor) was purchased from MedchemExpress (Monmouth Junction, NJ).

### Drug sensitivity assay

Cell viability was determined using Cell Counting Kit‐8 (Dojindo, Kumamoto, Japan) per the manufacturer's instructions. Cell viability was assessed 96 or 120 h after the indicated drug treatment. Three wells were used for each drug concentration, and the experiments were performed in triplicate. The half‐maximum inhibitory concentration (IC50) was calculated using Prism7 (GraphPad, La Jolla, CA) with a three‐parameter sigmoidal curve fit. The *P* values for the two‐curve comparisons were calculated using the extra sum of squares *F* test.

### Combination effect

The combination effect of two or three drugs was evaluated based on the combination index (CI) 19, 20 using Compusyn software (ComboSyn, Inc. Paramus, NJ). The combination effect was defined as follows: CI < 1 indicated a synergistic effect; CI = 1 indicated an additive effect; CI > 1 indicated an antagonistic effect.

### Gene expression analysis (DNA microarray)

Total RNA was extracted from H1299 parental and VR cell lines using an RNeasy Plus mini kit (Qiagen, Valencia, CA) per the manufacturer's instructions. RNA integrity was determined with an Agilent 2100 bioanalyzer (Agilent Technologies, Santa Clara, CA). The RNA was processed with the Ambion WT expression kit (Thermo Fisher Scientific K. K.), and GeneChip WT Terminal Labeling Kit (Affymetrix, Santa Clara, CA). These samples were hybridized to the GeneChip Human Gene 1.0 ST Array (Affymetrix), then washed, stained using the Fluidics Station 450 and scanned with the GeneChip Scanner 3000 (Affymetrix). The H1299 VR/H1299 parental cell expression ratio was calculated, and the differential expression of a gene was significant if its ratio exceeded 2. A pathway analysis was performed on the differentially expressed genes using GeneSpring GX (Agilent Technologies) and WikiPathways.

### Quantitative reverse transcription‐PCR (qRT‐PCR)

Total RNA from H1299 parental, H1299 VR, A549 parental, or A549 VR cells was reverse transcribed to cDNA using Ready‐To‐Go You‐Prime First‐Strand Beads (GE Healthcare Life Sciences, Pittsburgh, PA) per the manufacturer's instructions. For qRT‐PCR, each cDNA was diluted to 10 ng/*μ*L, and 2 *μ*L of cDNA was mixed with the THUNDERBIRD Probe qPCR Mix (Toyobo, Osaka, Japan) and TaqMan Gene Expression Assay probe/primer set (Thermo Fisher Scientific K. K.). The reactions were run using a StepOnePlus Real‐Time PCR System (Thermo Fisher Scientific K. K.). The comparative Ct (ΔΔCt) method was used to determine relative expression using *β*‐actin (*ACTB*) as the control gene 21. Each sample was run in triplicate. The following TaqMan probes were used: *ACTB* (Hs01060665_g1), integrin beta 3 (*ITGB3*, Hs01001469_m1), ATP‐binding cassette, subfamily B, member 1 (*ABCB1*, Hs00184500_m1), v‐src avian sarcoma viral oncogene homolog (*c‐SRC*, Hs01082246_m1), protein tyrosine kinase 2 (*FAK*, Hs01056457_m1), FYN (*FYN*, Hs00941600_m1), integrin‐linked kinase (*ILK*, Hs00177914_m1), and prominin 1 (*PROM1*,* CD133*, Hs01009257_m1).

### Western blot analysis

Total cell lysates were resolved by 4–15% SDS‐PAGE and transferred onto PVDF membranes. Primary antibodies to SFK, pTyr416 SFK, ITGB3, AKT, and pSer437 AKT were purchased from Cell Signaling Technology (Danvers, MA). ITGB1 and FAK were purchased from Merck Millipore (Darmstadt, Germany). FYN and pSer21 FYN were purchased from ABclonal Biotechnology (Woburn, MA). pTyr397 FAK was purchased from Santa Cruz Biotechnology (Santa Cruz, CA). *β*‐actin was purchased from Sigma‐Aldrich (St Louis, MO). Following an overnight incubation with the primary antibody, membranes were incubated with horseradish peroxidase‐conjugated secondary antibodies (Jackson ImmunoResearch, West Grove, PA), then visualized using the EzWestLumi Plus detection kit (Atto, Tokyo, Japan), and luminescence was detected using the LuminoGraph II imaging system (Atto).

### Drug efflux assay

ABCB1‐mediated drug efflux was measured using Cell‐Based Assay Calcein AM (Cayman Chemicals, Ann Arbor, MI) per the manufacturer's instructions and a previous report 22. The nuclei were counterstained with Hoechst 33342 (Dojindo). The fluorescence of each dye was assessed with an ArrayScan VTI (Thermo Fisher Scientific K. K.). Calcein fluorescence in the perinuclear ring was calculated using the HCS Studio 2.0 Client Software (Thermo Fisher Scientific K. K.).

### Gene silencing (siRNA)

The custom‐made annealed double‐strand siRNAs were purchased from Japan Bio Services Co., LTD (Saitama, Japan). The RNA sequences were as follows: si‐*c‐SRC*#1, forward GGUGUCUUAAUACUGUCCUTT, reverse AGGACAGUAUUAAGACACCTT; si‐*c‐SRC*#2, forward CCUUCCUGGAGGACUACUUTT, reverse AAGUAGUCCUCCAGGAAGGTT; si‐*FYN*#1, forward GAAAAAUUUCAAAUAUUGATT, reverse UCAAUAUUUGAAAUUUUUCTT; si‐*FYN*#2, forward CCCUGUACGGGAGGUUCACAAUCAATT, reverse UUGAUUGUGAACCUCCCGUACAGGGTT; si‐*ITGB3*#1 forward UGUGUGGAGUGUAAGAAGUTT, reverse ACUUCUUACACUCCACACATT; si‐*ITGB3*#2, forward CCAGAUGAUUCGAAGAAUUTT, reverse AAUUCUUCGAAUCAUCUGGCC; and si‐Control, forward GCGCGCUUUGUAGGAUUCGTT, reverse CGAAUCCUACAAAGCGCGCTT. Mission siRNA for human *ABCB1* were purchased from Sigma‐Aldrich (si‐*ABCB1*#1 SASI_Hs01_00087519, si‐*ABCB1*#2 SASI_Hs01_00087520). The siRNA (12.5 μl of a 20 μM solution) was transfected into VR cells that were approximately 60% confluent in 6‐well dishes with 5 *μ*L Lipofectamine 2000 and 500 *μ*L Opti‐MEM. For drug sensitivity assays, cells were detached from dishes with trypsin after 24 h of transfection, and 2,000 cells were seeded into each well of a 96‐well dish. After a 4‐h incubation, various VRB concentrations were added to the wells, and cell viability was determined after 120 h.

### Src‐Tyr416 immunohistochemistry of clinical specimens

Immunohistochemistry (IHC) was performed to measure pTyr416 SFK expression in operative FFPE tissue samples from sixty lung cancer patients who had undergone lung cancer resections and adjuvant VRB plus cisplatin chemotherapy between December 2002 and January 2007 at our institute. The characteristics and prognoses of these patients have been previously reported 23. The study protocol was approved by the ethics committee of our university (G0028‐5). The slides were stained with the pTyr416 SFK rabbit monoclonal antibody (Cell Signaling Technology) and VECTASTAIN Elite ABC HRP Kit (Vector Laboratories, Burlingame, CA) per the manufacturer's protocol. Each specimen was categorized as negative or positive. Time‐to‐event curves were estimated using the Kaplan–Meier method, and differences were evaluated with the log‐rank test. Statistical analyses were performed using JMP12 software (SAS Institute Inc. Cary, NC).

## Results

### Characteristics of VRB‐resistant cell lines

**Figure 1:**
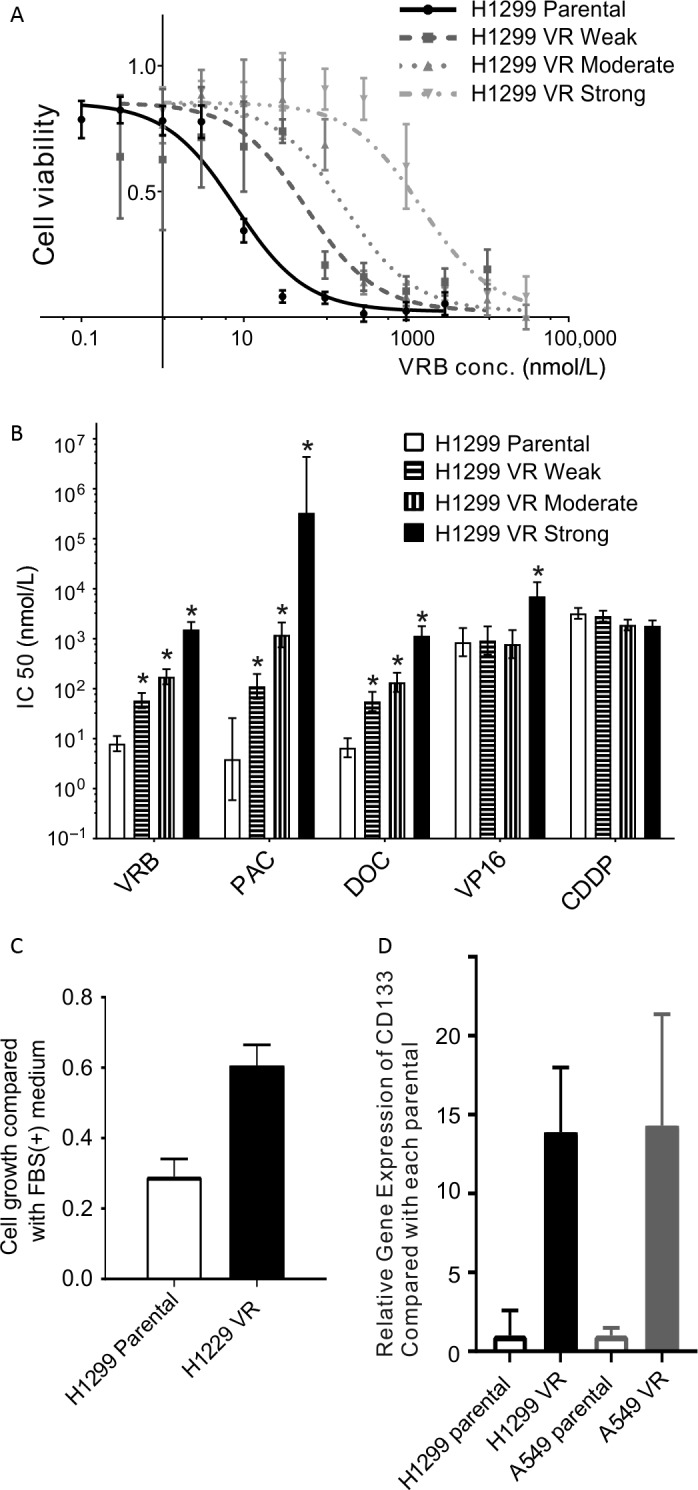
Characteristics of H1299 and A549 VR cells. (A) H1299 parental and VR (weak, moderate, and strong resistant) cell viabilities in response to VRB. H1299 parental and VR cells were treated for 96 h with increasing concentrations of VRB. Cell viability was determined using the WST‐8 assay and is shown as a percentage of the value of the untreated cells. The sigmoid curves were drawn using Prism software. Experiments were performed in triplicate. (B) The IC50 values for VRB, PAC, DOC, VP‐16, and CDDP in H1299 parental and VR cells. Cell viability in response to PAC, DOC, VP‐16, and CDDP was measured as described for VRB. Experiments were performed in triplicate. The IC50 values were calculated using Prism software. The error bars show the 95% CI. * *P* < 0.05 relative to the control (extra sum of squares *F* test). PAC, paclitaxel; DOC, docetaxel; VP‐16, etoposide; CDDP, cisplatin. (C) H1299 parental and VR cell growth rates in FBS‐free medium. H1299 parental and VR cell viabilities were determined after 96‐h incubations in FBS‐free medium. Results are shown as the ratio to cells that were grown in FBS‐containing medium and as the mean ± SEM. (D) Relative mRNA expression of CD133 in H1299 and A549 VR cells. Results are shown as the fold change of CD133 expression relative to the corresponding parental cell line and as the mean ± 95% CI.

The VRB IC50 for the VR cells exceeded 100 times that for the parental cells (Fig. 1A). H1299 VR cells had a cross‐resistance to paclitaxel (PAC), docetaxel (DOC), and etoposide (VP‐16) and were sensitive to cisplatin (CDDP) (Fig. 1B). The H1299 VR cell growth rate in fetal bovine serum (FBS)‐free medium compared with that in FBS‐containing medium was higher than that of the H1299 parental cells (Fig. 1C). A qRT‐PCR analysis of a cancer stemness marker, CD133 24, showed significant upregulation compared with the corresponding parental cells (Fig. 1D). But, no other generally accepted cancer stemness markers were not significantly upregulated.

### Gene expression comparison of parental versus VR cells by microarray and qRT‐PCR

**Figure 2:**
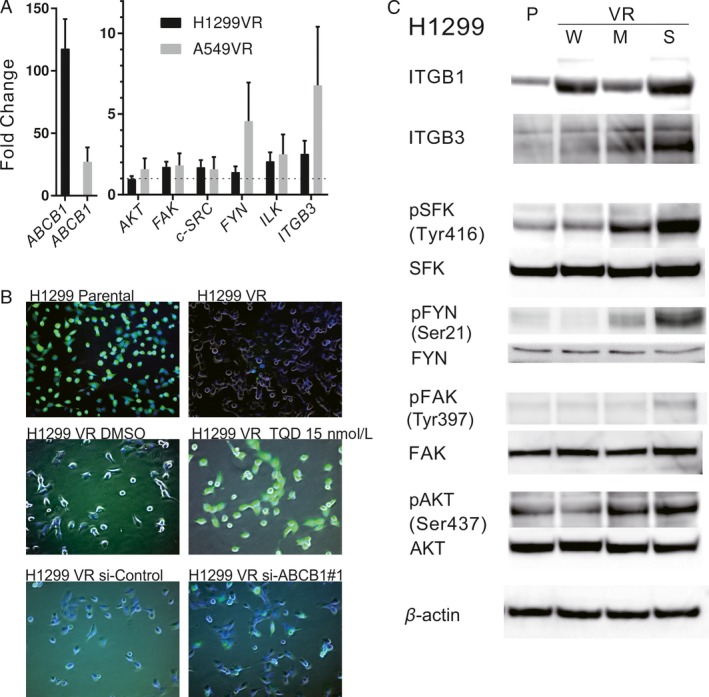
Gene and protein expression comparisons for parental versus VR cells. (A) Relative mRNA expression of *ABCB1*,* AKT*,* FAK*,* c‐SRC*,* FYN*,* ILK*, and *ITGB3* in H1299 and A549 VR cells. Results are shown as the fold change in gene expression relative to the corresponding parental cell line and as the mean ± 95% CI. (B) Calcein fluorescence in H1299 parental and VR cells. After a 30‐min incubation with tariquidar (TQD) or DMSO, Calcein AM was added to the cells. After 30 min, fluorescent images were obtained with the BZ‐9000 (Keyence Corporation, Osaka, Japan). Nuclei were counterstained with Hoechst 33342. Images were merged using ImageJ. In H1299 VR si‐ABCB1#1 and si‐Control, transfection of siRNA was done 120 h before. Calcein (green), Hoechst 33342 (blue), and phase contrast images (gray) are shown.(C) Western blot analysis of whole‐cell lysates from H1299 parental and VR (W: Weak, M: Moderate and S: Strong resistant) cells. Membranes were blotted with total ITGB1, ITGB3, pTyr416 SFK, total SFK, pSer21 FYN, total FYN, pTyr397 FAK, total FAK pSer437 AKT, and total AKT antibodies; *β*‐actin was used to confirm equal protein loading.

A microarray‐based comparison of H1299 parental and H1299 VR cells revealed that 205 of 23,230 genes were highly expressed (fold change >2) in H1299 VR cells. ABCB1 was the most highly expressed gene in H1299 VR cells and a pathway analysis of the 205 genes indicated that the FA pathways were significant (*P* = 0.00086). High expression of these genes was confirmed by qRT‐PCR (Fig. 2A).

### Activation of drug efflux and FA pathway in H1299 VR cells

Efflux assays revealed an enhancement of ABCB1‐mediated drug efflux in H1299 VR cells (Fig. 2B). And drug efflux in H1299 VR cells was reduced both by an ABCB1 inhibitor, tariquidar (TQD), or ABCB1 silencing, respectively (Fig. 2B).

We subsequently examined the expression and activation levels of focal adhesion‐related proteins by Western blot. Integrins *β*1 and *β*3 were highly expressed in VR cells relative to parental cells. The high expression of pTyr416 SFK, pSer21 FYN, pTyr397 FAK, and pSer437 AKT indicated FA pathway activation in VR cells (Fig. 2C). Integrin *β*3, pTyr416 SFK, and pSer21 FYN expression incrementally increased with the VR resistance level.

### Activation of SFK in human lung cancer samples

**Figure 3:**
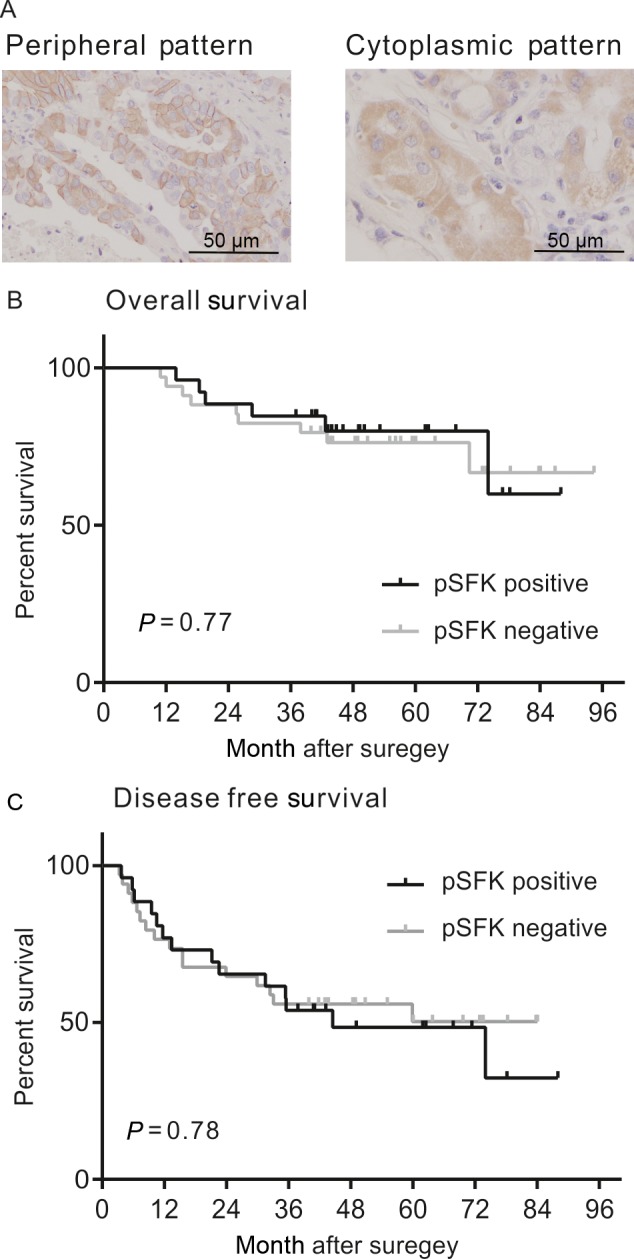
pSFK expression in human lung cancer samples and its correlation with survival. (A) Representative Immunohistochemistry images of lung adenocarcinoma sections with anti‐pTyr416 SFK. Tumor cells show peripheral (left) or cytoplasmic staining (right).(B, C) Kaplan–Meier curves for overall survival and disease‐free survival per pTyr416 SFK expression. Log‐rank *P* values were calculated using JMP software.

The patient characteristics are shown in the Table 1. The cancer cells were stained with a pTyr416 SFK antibody in a peripheral or cytoplasmic manner (Fig. 3A). Of the 60 operative samples, 34 samples were negative, and 26 samples were positive. The patient prognoses for each group relative to pTyr416 SFK expression are shown in Figure 3B; the pTyr416 SFK expression status did not show a correlation with patient prognosis. Furthermore, the staining pattern (peripheral or cytoplasmic) did not affect survival (data not shown).

**Table 1:** Characteristics of the patients included in this study

Characteristic	Number of patients (n = 60)
Age (years)	
Median	64
Range	40–75
Gender (%)	
Male	40 (67)
Female	20 (33)
Tumor histology (%)	
Adenocarcinoma	34 (57)
Squamous cell carcinoma	21 (35)
Large cell carcinoma	3 (5)
Adenosquamous cell carcinoma	1 (2)
Pleomorphic carcinoma	1 (2)
Pathological stage (UICC, 7th edition) (%)	
Stage IIA	21 (35)
Stage IIB	8 (13)
Stage IIIA	31 (52)
Patients who completed cycles (%)	
Cycle 1	58 (97)
Cycle 2	55 (92)
Cycle 3	50 (83)
Cycle 4	28 (47)

### The effect of ABCB1 and SFK knockdown by siRNA on VRB sensitivity

**Figure 4:**
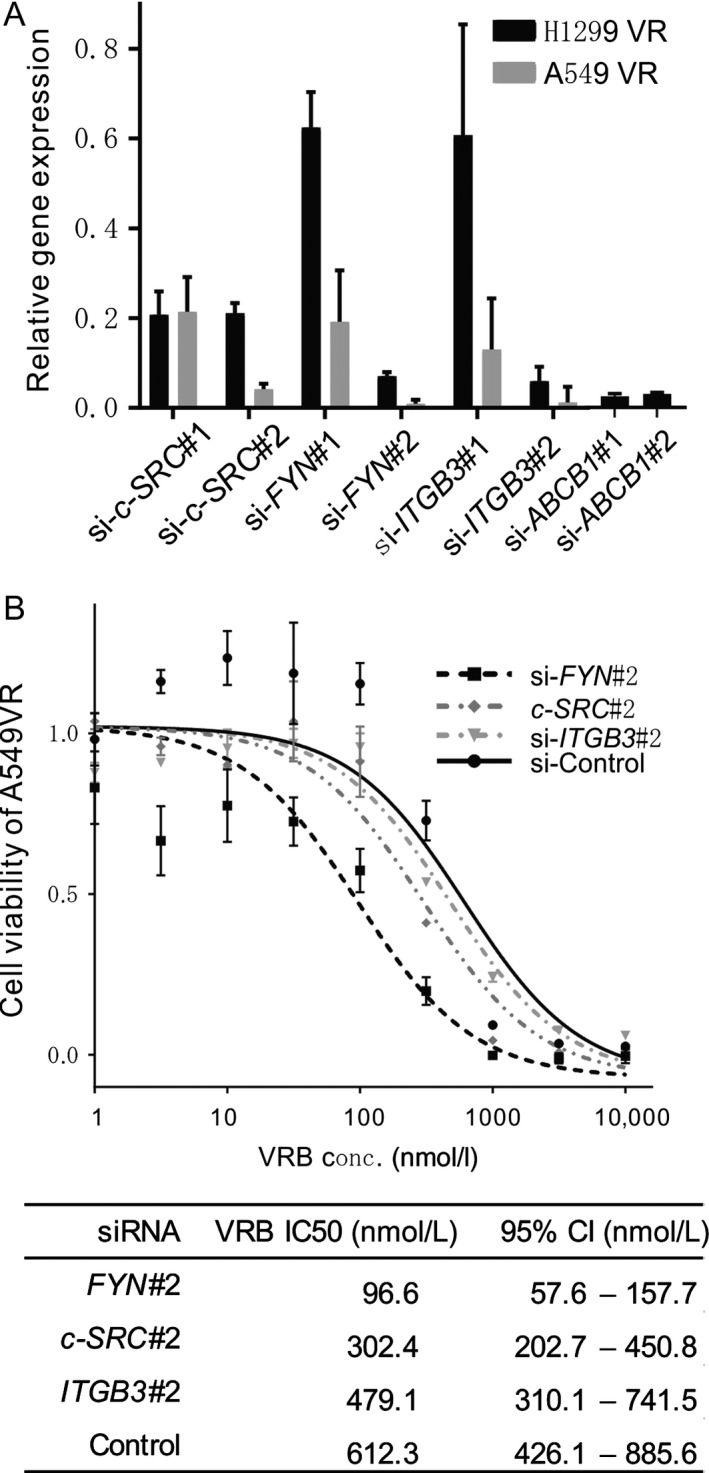
*c‐SRC*,*FYN*, and *ITGB3* silencing by siRNA and its effect on VRB sensitivity. (A) The *c‐SRC* gene in H1299 VR and A549 VR cells was knocked down with siRNA transfections (si‐*c‐SRC*#1 and si‐*c‐SRC*#2). The inhibitory effects on *c‐SRC* gene expression were measured by qRT‐PCR. The relative mRNA expression of *c‐SRC* in si‐*c‐SRC*#1‐ or si‐*c‐SRC*#2‐transfected cells is shown as the fold change in *c‐SRC* expression relative to the corresponding si‐Control cell line and as the mean ± 95% CI. The inhibitory effects of the *FYN* (si‐*FYN*#1 and si‐*FYN*#2), *ITGB3* (si‐*ITGB3*#1 and si‐*ITGB3*#2), or *ABCB1* (si‐*ABCB1*#1 and si‐*ABCB1*#2) gene silencing are also shown. (B) A549 VR siRNA‐transfected cell viability. A549 VR cells that were transfected with siRNA (*c‐SRC*#2, *FYN*#2, *ITGB3*#1, or Control) were treated for 120 h with increasing concentrations of VRB. The data from the cell viability assay (WST‐8 assay) are expressed as a percentage of the value of the untreated cells. The IC50 was calculated using Prism software. Experiments were performed in triplicate.

The knockdown efficiency was determined by qRT‐PCR using RNA that was extracted from the transfected cells 48 h after the transfection (Fig. 4A). Effects on protein expression were also assessed in si‐*ABCB1*#1 treated cells (120 h after transfection, Fig. S1A). We used si‐*ABCB1*#1, si‐*c‐SRC*#2, si‐*FYN*#2, and si‐*ITGB3*#2 for further experiments. The A549 VR cells showed more effective inhibition than the H1299 VR cells. The VRB IC50 for the si‐*ABCB1*#1 treated H1299 VR cells was decreased, however, it was not fully recovered to that of parental cells (Fig. S1B). The VRB IC50 for the si‐*FYN*#2‐treated A549 VR cells was significantly decreased compared with that of the control (*P* = 0.0002). On the other hand, *c‐SRC* and *ITGB3* silencing did not show prominent VRB IC50 decreases (Fig. 4B). These results indicate that SFK (specifically FYN) plays pivotal roles in VRB resistance. However, the knockdown of *FYN* in the H1299 VR cells did not significantly restore VRB sensitivity (Fig. S1C).

### Effect of ABCB1, SFK, and an integrin inhibitor on VR cells

**Figure 5:**
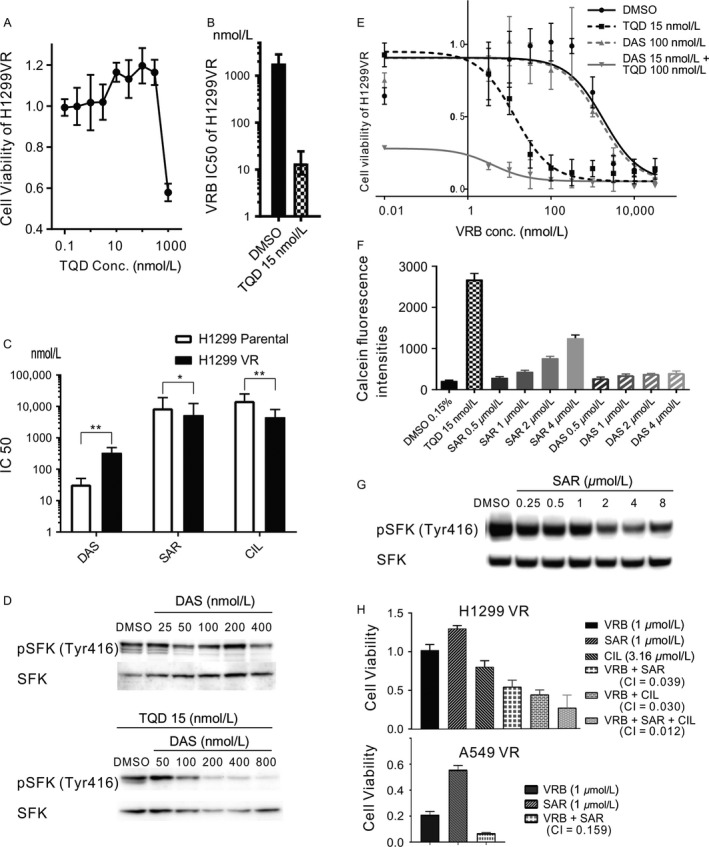
Recovery of VRB sensitivity through ABCB1 or SFK inhibition. (A) Cell viability of H1299 VR cells treated with tariquidar. H1299 VR cells were treated for 96 h with increasing concentrations of tariquidar. Cell viability is shown as a percentage of the value of the untreated cells. The IC50 was not calculated due to the lack of low viability data. (B) The IC50 values for VRB in H1299 VR cells that were treated with DMSO or tariquidar. H1299 VR cells were treated for 96 h with increasing concentrations of VRB and 15 nmol/L tariquidar or DMSO. The IC50 values were calculated using Prism software. The error bar shows the 95% CI. (C) IC50 values for dasatinib, saracatinib, and cilengitide in H1299 parental and VR cells. Cells were treated for 96 h with increasing concentrations of dasatinib, saracatinib, or cilengitide. The IC50 was calculated using Prism software. * *P* < 0.05, ** *P* < 0.01. (D) Western blot analysis of whole‐cell lysates from H1299 VR cells. Cells were treated with dasatinib or dasatinib plus tariquidar for 3 h, and cell lysates were collected. Membranes were blotted with the pTyr416 and total SFK antibodies. (E) Cell viability in response to VRB for H1299 VR cells that were treated with tariquidar and/or dasatinib. Cells were treated for 96 h with increasing concentrations of VRB plus 100 nmol/L dasatinib and/or 15 nmol/L tariquidar. The cell viabilities from the WST‐8 assays are expressed as the percentage of the value for untreated cells, and the sigmoid curves were drawn by Prism software. Determinations were performed in triplicate. (F) Calcein fluorescence intensities in H1299 VR cells. Calcein fluorescence after incubations with tariquidar, saracatinib, dasatinib, or DMSO was detected with ArrayScan VTI. Perinuclear fluorescence was quantified using HCS Studio 2.0 Client Software. (G) Western blot analysis of whole‐cell lysates from H1299 VR cells. Cells were treated with saracatinib for 3 h. Membranes were blotted with the pTyr416 and total SFK antibodies. (H) Cell viability of H1299 VR and A549 VR cells that were treated with 1 *μ*mol/L VRB and/or 1 *μ*mol/L saracatinib and/or 3.16 *μ*mol/L cilengitide. The data from the cell viability assays (WST‐8 assay) are expressed as a percentage of the value for the untreated cells. The combination index (CI) was calculated using Compusyn software. TQD, tariquidar; DAS, dasatinib; SAR, saracatinib; CIL, cilengitide.

Although a 96‐h exposure to 300 nmol/L tariquidar alone did not produce H1299 VR cell toxicity (Fig. 5A), H1299 VR cells that were treated with as little as 15 nmol/L tariquidar recovered their VRB sensitivity (Fig. 5B).

We subsequently aimed to inhibit SFK activation using dasatinib (DAS), an SFK inhibitor. However, the H1299 VR cells had cross‐resistance to dasatinib (Fig. 5C), and 100 nmol/L dasatinib did not effectively inhibit SFK activation (Fig. 5D) nor was it effective toward VRB sensitivity in H1299 VR cells (Fig. 5E). Because dasatinib is also a substrate for ABCB1 25, we combined tariquidar and dasatinib to inhibit efflux and improve the effect of dasatinib. By combining 15 nmol/L tariquidar with 100 nmol/L dasatinib, we effectively inhibited SFK activation and reduced H1299 VR cell survival. The combination of 100 nmol/L VRB with 100 nmol/L dasatinib plus 15 nmol/L tariquidar almost completely inhibited cell survival (Fig. 5D and E).

We next used saracatinib (SAR), a specific inhibitor of SFK that also inhibits ABCB1 26. The efflux assay revealed a concentration‐dependent ABCB1‐mediated calcein efflux inhibition (Fig. 5F). Consistent with these results, H1299 VR cells did not show cross‐resistance to saracatinib; the IC50 for saracatinib in H1299 VR cells was notably lower than that in H1299 parental cells (Fig. 5C). The effective inhibitory dose for ABCB1 (2 *μ*mol/L saracatinib) also remarkably inhibited SFK activity (Fig. 5G). The 1 *μ*mol/L saracatinib plus 1 *μ*mol/L VRB combination more effectively inhibited H1299 VR and A549 VR cell viability than saracatinib or VRB alone. The combination index (CI) values for VRB plus saracatinib in the H1299 VR and A549 VR cells indicated that the concomitant use of VRB and saracatinib had synergistic effects on the VR cells (Fig. 5H).

Then we tested the effectiveness of cilengitide (CIL), an integrin *α*v*β*3 inhibitor. Because cilengitide, a cyclic RGD pentapeptide, targets the extracellular domain 27, we predicted that ABCB1 activation would not alter its effectiveness. As expected, the IC50 of cilengitide in H1299 VR cells was significantly lower than the IC50 in H1299 parental cells (Fig. 5C). The combination index for VRB plus cilengitide and cilengitide, VRB plus saracatinib showed synergism in H1299 VR cells (Fig. 5H).

## Discussion

VRB is a common chemotherapeutic agent in lung cancer therapies, particularly in postoperative chemotherapy. However, its narrow applicability to other cancer types has limited the number of reports that address VRB resistance relative to other drugs. Several reports have shown that SFK inhibition potentiates the anticancer activity of paclitaxel 28, 29. Although VRB and paclitaxel belong to different drug families, both drugs affect the same target, the microtubule. It is conceivable that VRB has a similar resistance mechanism to that of paclitaxel. Forest et al. 30 reported decreased activation of paxillin in A549 cells that were cocultured with VRB; the dephosphorylation of paxillin functions as an apoptotic signal. Consistently, SFK activation, which activates paxillin, results in cell survival.

Although v‐Src, which contains a truncating mutation among its regulatory C‐terminal tyrosine residues, is an oncogene, SFK is prevalent in tumor progression and in maintaining the neoplastic phenotype; it is not involved in tumor initiation or growth 31. The SFK function in cancer cells is independent of its mutation status, c‐SRC activation by oncogenic mutations has not been detected in most cancers 32. In the context of acquired resistance, de novo mutations would not be the main cause of chemo‐resistance because clinically relevant mutations after chemotherapy are reportedly rare 33. The phosphorylation status of SFK is controlled by the functional or activation changes of its regulatory proteins, such as integrins or receptor tyrosine kinases (RTKs). We focused on integrin *β*3 as an upstream regulator of SFK because VR cells showed high integrin *β*3 expression. While the *ITGB3* knockdown did not alter VRB sensitivity in VR cells, cilengitide showed an inhibitory effect on VR cells. These results indicate that integrin *β*3 is not an independent activator of SFK; other transmembrane proteins, such as integrins *α*v*β*5 and *α*5*β*1, might also affect SFK activation in VR cells, because cilengitide targets integrins *α*v*β*3, *α*v*β*5, and *α*5*β*1 27. Moreover, SFK interacts with numerous genetic and signaling pathways, including the EGFR, Janus‐activated kinase (JAK)/signal transducers and activators of transcription (STAT), and vascular endothelial growth factor (VEGF) pathways 32. Further studies are needed to elucidate the roles of these interactions in VRB resistance.

SFK proteins are comprised of nine family members—c‐Src, Yes, Fyn, Lyn, Lck, Hck, Fgr, Blk, and Yrk. In this study, *FYN* expression was higher than *c‐SRC* expression, and *FYN* knockdown restored VRB sensitivity in A549 VR cells; *c‐SRC* knockdown showed little effect. This result suggested a higher importance for FYN in VRB resistance, although technical problems may have influenced the results. Specifically, our siRNA for *FYN* (si‐*FYN*#2) inhibited gene expression more effectively than si‐*c‐SRC*#2. As we only examined the *c‐SRC* and *FYN* knockdown according to both the high expression of the Fyn gene in VR cells and the previous report which showed the effect of *c‐SRC* and *FYN* on EGFR‐TKI sensitivity 34, the differences between these family members were not well described.

Results of our report showed difference in effect of *FYN* knockdown between H1299 VR and A549 VR cells. Depending on the other results of FA pathway activation and SFK inhibitors, we considered FYN also have an important role in VRB resistance in H1299 VR cells, however, the effect of siRNA was limited because the cells with higher growth rate, like H1299, showed lower efficiency in gene knockdown 35, 36.

In addition to SFK, SFK inhibitors have multiple targets, such as Abl, EGFR, PDGFR, and c‐Kit (Table S1). Among these targets, inhibition of EGFR should affect the survival of VRB‐treated cancer cells, because Pirker et al. reported longer survival in addition of cetuximab to CDDP plus VRB 37. The effects of dasatinib or saracatinib on EGFR inhibition are not described in this report, but those effects seem to be limited because both H1299 and A549 do not have EGFR mutation. We used saracatinib to inhibit both SFK and ABCB1, which are the two main factors in VRB resistance. Saracatinib may be a strong candidate drug for patients with relapse after VRB therapy. However, at 2 *μ*mol/L saracatinib, which effectively inhibited SFK and ABCB1 activities in VR cells, the drug concentration was remarkably higher than the serum concentration in patients from a previous study who took once‐daily 175‐mg doses of saracatinib 38. This finding may be a causative factor in the negative clinical trial results for NSCLC 39 and other malignancies 40, 41, 42, 43.

Our IHC results revealed no significant correlation between pSFK (Tyr416) expression and patient prognosis. Zhang et al. reported that pSFK expression was not associated with the pathological disease stage or survival in patients with stage I‐II NSCLC who had undergone lung resections 44. Laurie et al. reported no correlation between Src protein expression and patient outcome in a phase II trial of saracatinib in previously treated advanced NSCLC patients; phospho‐Src was not assessed 39. These reports claimed that pSFK expression was not a clinically valuable biomarker for prognostic predictions. According to our results, phospho‐FYN expression may predict the prognosis, but we could not find reliable phospho‐Fyn specific antibody for IHC. Additionally, our results revealed post‐VRB treatment SFK activation. However, our clinical samples were obtained during the surgeries of patients who had not received chemotherapy or radiotherapy. These samples did not reflect the acquisition of postchemotherapy VRB resistance. However, we had better know the information whether patient's tumor has innate chemo‐resistance (not acquired resistance) before treatment, especially in the setting of postoperative adjuvant chemotherapy.

Cancer stem cells are a reported cause of drug resistance and poor prognosis 45. The VR cells in our study show several characteristics that are associated with cancer stemness. Desgrosellier et al. reported that the integrin *α*v*β*3–Src unit promotes anchorage‐independence^46^. Integrin *β*3 also drives tumor stemness 15. We previously reported that CD133, a cell surface marker that is used to isolate cancer stem cells, is a statistically significant factor for predicting a poor lung adenocarcinoma prognosis 24, and Su et al. reported that CD133 activates integrin‐Src‐Akt signaling 47. Although our study did not address the relationship between SFK‐related drug resistance and cancer stemness, it is possible that the acquisition of drug resistance and cancer stemness share the same root cause and that SFK may be a target for eradicating cancer stem cells.

In conclusion, we have described both of the ABCB1 overexpression and activation of the FA pathway, and its availability for inhibition in VRB‐resistant cells. Moreover, saracatinib and cilengitide are particularly promising inhibitors of ABCB1‐accelerated cells. However, several discrepancies between the laboratory results and clinical outcomes remain. Further studies are needed to identify clinically applicable target drugs and biomarkers that will improve disease prognoses and predict the therapeutic efficacy of SFK inhibition.

## Conflict of interest

None of the authors have any conflicts of interest to disclose regarding this study.

## Supporting information


**Figure S1**. ABCB1 protein expression in H1299 parental, VR, and each si‐*ABCB1*#1 treated cells.

**Table S1.** Inhibitory activity of AZD0530 (saracatinib) on cell line proliferation. IC50 values are the mean of at least three measurements.
