# Supplementary material for: The synergistic role of ATP‐dependent drug efflux pump and focal adhesion signaling pathways in vinorelbine resistance in lung cancer
Source: Cancer Med. 2018 Jan 10;7(2):408–19. doi: 10.1002/cam4.1282 (PMC5806107; doi:10.1002/cam4.1282)
Supplement: Supplementary file 2 — Table S1. Inhibitory activity of AZD0530 (saracatinib) on cell line proliferation. IC50 values are the mean of at least three measurements. [file CAM4-7-408-s002.docx]

Supplemental Table 1. Inhibitory activity of AZD0530 (saracatinib) on cell line proliferation.

IC50 values are the mean of at least three measurements

| Kinase | Definition | Mean IC50, nM |
| --- | --- | --- |
| c-Src | Ubiquitous Src family member | 2.7 |
| Lck | Immune cell-restricted Src family member | <4 |
| c-Yes | Ubiquitous Src family member | 4 |
| EGFR L861Q | Activating mutation of epidermal growth factor receptor | 4 |
| Lyn | Immune cell-restricted Src family member | 5 |
| EGFR L858R | Activating mutation of epidermal growth factor receptor | 5 |
| Fyn | Ubiquitous Src family member | 10 |
| Fgr | Immune cell-restricted Src family member | 10 |
| Blk | Immune cell-restricted Src family member | 11 |
| v-Abl | Viral Abelson tyrosine kinase | 30 |
| EGFR | Epidermal growth factor receptor tyrosine kinase | 66 |
| c-kit | Stem cell factor receptor tyrosine kinase | 200 |
| EphA2 | Ephrin receptor tyrosine kinase | 236 |
| Csk | c-terminal Src kinase (negative regulator of Src) | >1000 |
| PDGFRβ | Platelet-derived growth factor receptor tyrosine kinase | >5000 |
| PDGFRα | Platelet-derived growth factor receptor tyrosine kinase | 10,000 |
| CDK2 | Cyclin-dependent kinase 2 | 10,000 |
| Flt-4 (VEGFR3) | Fms-like tyrosine kinase 4 | >10,000 |
| FGFR (FGFR1) | Fibroblast growth factor receptor tyrosine kinase | >10,000 |
| AUR-3 | Aurora kinase-3 | >10,000 |
| MEK | Mitogen-activated protein kinase | 14,000 |
| KDR (VEGFR2) | Kinase insert domain-containing receptor | 21,000 |
| Flt-1 (VEGFR1) | Fms-like tyrosine kinase 1 | >100,000 |

Quoted and modified from Green et al. Mol Oncol. 2009.

Green T, Fennell M, Whittaker R, Curwen J, Jacobs V, Allen J, et al. (2009). Preclinical anticancer activity of the potent, oral Src inhibitor AZD0530. Mol Oncol 3: 248–261.
